# Mr. Crochford, on the Efficacy of Vacciola

**Published:** 1804-09-01

**Authors:** W. Crochford

**Affiliations:** Lewes


### *To the Editors of the Medical and Physical Journal.*

GENTLEMEN,

DOUBTS having lately been expressed, that vaccine inoculation will not prevent small-pox for more than three years, I beg the insertion of the following cases in your useful Journal, as the first three of them shew that the preventive power of vaccine inoculation, at least, exceeds four years, and am persuaded ought to be considered as permanent.

| | | | |
|---|---|---|---|
| 1 | Maria Fuller, | aged 10 years | Inoculated with Cow-Pock in May, 1800. |
| 2 | John Fuller, | aged 8 years | |
| 3 | Edwin Fuller, | aged 6 years | |
| 4 | William Fuller, | aged 4 years, | ditto April, 1803. |
| 5 | Sarah Ann Fuller, | aged 16 months, | ditto March, 1804. |

The cow-pock in all run the usual regular course.

The parents wished the above children to be inoculated with variolous matter as a *test* of the efficacy of the first inoculation. Accordingly, this was done June 5, 1804.

The

June 9, evident marks of inflammation in the arms of all.

June 13, the inflammation on the arm of Sarah Anne gone; on those of Maria and Edwin subsiding, but the inoculated parts contained matter; two days later there only remained on each a dry scab. In John and William the inflammation on the arms was more considerable, and lasted some days longer: on the arm of John, very near to the part inoculated, two small pimples appeared, but no eruptions besides. William had two small pimples on the lower arm and one on the cheek, which remained out a few days, but never contained a fluid.

In Edwin and Ann there was no perceptible indisposition.

Maria, John, and William had some stiffness under the arm, and a slight degree of feverish heat on the seventh and eighth days after inoculation; John had the most, the inflammation at the part inoculated being the greatest. The above cases were shewn to several medical gentlemen, who all agreed that the prior cow-pock inoculation had effectually prevented small-pox.

Thomas Fuller, aged twelve years, a brother of the above children, had been inoculated with small-pox matter above ten years ago; and though he had then no eruption of pustules, was supposed to have had the disease; he had afterwards been repeatedly allowed to go near persons affected with confluent small-pox with impunity. He was again inoculated with small-pox matter at the particular request of his mother, at the same time and from the same source (viz. June 5, 1804) as the above children. He was seized with feverish symptoms on the sixth day, and had a copious eruption of small-pox pustules over the face and body on the eighth day, which run the usual course, and left several marks behind. This last case demonstrates that the variolous matter employed for inoculation, was sufficient to have produced small-pox in all, had not the previous vaccine inoculation prevented it. The slight febrile symptoms on the seventh and eighth days, which occurred in those who had had cow-pox, may, I conceive, fairly be attributed to local irritation.

I am, &c.

*Lewes, August* 12, 1804.  W. CROCHFORD.

